# Mycotoxin contamination in moldy slices of bread is mostly limited to the immediate vicinity of the visible infestation

**DOI:** 10.1016/j.fochx.2024.101563

**Published:** 2024-06-18

**Authors:** Nicole Ollinger, Alexandra Malachova, Michael Sulyok, Rudolf Krska, Julian Weghuber

**Affiliations:** a FFoQSI – Austrian Competence Centre for Feed and Food Quality, Safety & Innovation, Stelzhamerstr. 23, 4600 Wels, Austria; b FFoQSI – Austrian Competence Centre for Feed and Food Quality, Safety & Innovation, Konrad Lorenz Str. 20, 3430 Tulln, Austria; c Department for Agrobiotechnology (IFA-Tulln), University of Natural Resources and Life Sciences, Vienna (BOKU), Konrad Lorenz Str. 20, 3430 Tulln, Austria; d Institute for Global Food Security, School of Biological Sciences, Queen's University Belfast, University Road, Belfast, BT7 1NN, Northern Ireland, United Kingdom; e University of Applied Sciences Upper Austria, Stelzhamerstrasse 23, 4600 Wels, Austria

**Keywords:** Mycotoxins, DNA barcoding, Mold species, Bread, Targeted metabolite profiling

## Abstract

Bread is an important staple food that is susceptible to spoilage, making it one of the most wasted foods. To determine the safety of partially moldy bread, five types of bread were inoculated with common mold species. After incubation, the metabolite profile was determined in and under the inoculation spot, as well as at a lateral distance of 3 cm from the moldy spot. The result showed that the metabolites were exclusively concentrated in the inoculation area and directly below the inoculation area. The only exception was citrinin, a mycotoxin produced by *Penicillia* such as *Penicillium citrinum*, which was detected in almost all tested bread areas when inoculated with the corresponding strains. The results of our study suggest that the removal of moldy parts may be a solution to reduce food waste if the remaining bread is to be used, for example for insect farming to produce animal feed.

## Highlights

• Mycotoxins such as fumonisins and roquefortine C were only detectable in moldy bread close to the inoculation spot except for citrinin.
• Identification of undesired mold species by DNA barcoding revealed contamination by six common mold species.
• Regulations for the recycling of moldy bread are required

## 1 Introduction

Bread culture has a long historical tradition in Europe. Each country and even each region has its own bread types, which vary in grain type, fermentation method, and preferred oven for baking, resulting in different sweetness, spiciness, aroma, texture, flavor, and appearance. Due to the different ingredients and textures, the shelf life of the bread types can also vary (Axel et al., 2017; Giménez et al., 2013; Melini & Melini, 2018; Milani & Heidari, 2017).

According to the Statista Research Department, the per capita bread consumption in Austria was 40 kg in 2022 and is expected to increase to 41.1 kg in 2024. Worldwide, the average bread consumption per person was 23.5 kg in 2022 and is expected to be 24.8 kg in 2024 (Statista, 2024). Therefore, bread is one of the most important staple foods worldwide. Depending on the type of bread and its composition, it tends to go moldy quite quickly.

In a previous study, the distribution of mycotoxins released by mold on a stack of sliced bread was investigated. The top slice, which was heavily moldy, contained high levels of mycotoxins released by *Chaetomium globosum* and *Penicillium chrysogenum*, and was no longer suitable for human consumption. The second slice was slightly moldy and contained fewer secondary metabolites. For example, in the top slide, the center of the *Penicillium chrysogenum* spot contained 716 ng roquefortine C per 1 g of bread. The value was reduced in the second slice on the same position down to 15.2%. The third slice was almost free of mycotoxins except for those evenly distributed throughout the samples due to contamination from the grain used to bake the bread (Ollinger et al., 2022). This paper raised many discussion points regarding the safety of bread. The most important questions were (a) whether it is safe to cut off the moldy part of the bread and eat the rest, (b) whether the type of bread is relevant for the distribution of mycotoxins or other fungal metabolites and (c) whether it is beneficial if the bread is already sliced by the industry or whether it is better to sell it as a whole loaf.

However, given that it is generally not recommended that people consume partially moldy bread, it can either be used for biofuel production (Narisetty et al., 2022; Pietrzak & Kawa-Rygielska, 2015) or, at least the non-moldy parts could eventually be used for other purposes. For example, as feed for insect farming, as some insects may be able to break down mycotoxins in their metabolism. The demand for food and feed alternatives increases, so does the production of insects either as food or preferably feed. This seems to be a very realistic scenario, but it needs to be further studied which insects are resistant and which are sensitive to mycotoxins. In this context it has been reported that the larvae of the lesser mealworm (*Alphitobius diaperinus*) and the black soldier fly (*Hermetia illucens*) larvae have the ability to degrade or at least not accumulate mycotoxins. High concentrations of 0.5 mg/kg aflatoxin B1, 125 mg/kg deoxynivalenol, 2.5 mg/kg ochratoxin A or 12.5 mg/kg zearalenone were applied, which is 25 times the level permitted for complete feed (Jensen & Nørgaard, 2022).

In contrast, the mealworm *Tenebrio molitor*, which is suitable for human consumption, shows impaired growth and locomotor activity in the presence of 25 mg/kg deoxynivalenol (Janković-Tomanić et al., 2019). The larvae of the flour pests *Tribolium confusum, Lasioderma serricorne* and *Attagenus megatoma* were growth inhibited by citrinin and ochratoxin A (Wright et al., 1980). Common food relevant citrinin producers are *Penicillium expansum*, *Penicillium citrinium*, *Penicillium verrucosum* and *Monascus* species, but a number of other species are also known to produce this compound (EFSA, 2012). These and other publications (Evans & Shao, 2022) prove, that the safety of feeding insects with moldy food depends on the type of mycotoxin, the concentrations presented and the insect species to grow on the substrate. Eating insects is very common in various regions of the world, although in Europe the willingness to eat insects is limited and therefore not an option at the moment. In a recent study in Germany, it was shown that people are still very reluctant to eat insects due to psychological barriers to consumption, such as disgust and food neophobia. After trying it once, they were more open-minded (Orsi et al., 2019). However, using insects as animal feed could also be a novel approach to find a suitable use for spoiled bread, especially as there is evidence (see above) that insects are able to detoxify mycotoxins or at least not ingest them.

The work reported here aims to provide insight into the safety considerations associated with partially moldy bread and a better understanding of the mycotoxin production of five mold species growing on five types of bread and the distances over which mycotoxins can diffuse. It is a follow-up to an earlier paper on moldy bread, which raised questions such as (a) is it safe to cut off the moldy part of the bread and eat the rest, (b) is the type of bread relevant for the distribution of mycotoxins or other fungal metabolites, and (c) is it beneficial if the bread is already sliced by the industry or is it better to sell it as a whole loaf. *Paecilomyces variotii*, *Penicillium citrinum*, *Penicillium chrysogenum*, *Aspergillus niger* and *Chaetomium globosum* were inoculated on wheat/rye/oat sourdough toast bread, white wheat toast bread, sliced wheat/rye bread, whole loaf wheat/rye bread, and gluten-free corn bread. The toast breads containing preservatives showed a slower mold growth than the preservative-free breads. The diffusion distances of the mycotoxins were very short in all breads with the exception of citrinin, which was detected at high amounts in almost every bread aliquot measured, as soon as citrinin is produced by the mold.

## 2 Material and methods

### 2.1 Chemicals, cultures, and bread samples

Chemicals and culture medium were obtained from Sigma Aldrich (Schnelldorf, Germany) unless otherwise stated. Potato dextrose agar from VWR (Darmstadt, Germany) was diluted and autoclaved at 121 °C for 20 min and poured warm into 9 cm sterile petri dishes. Agar plates were allowed to polymerize at room temperature for at least 4 h and kept at 4 °C until usage.

Lyophilized cultures of *Paecilomyces variotii* (CECT 20360), *Penicillium citrinum* (CECT 2274), *Penicillium chrysogenum* (CECT 2307) and *Aspergillus niger* (CECT 2088) were purchased at the Spanish Type Culture Collection (Paterna, Spain) and *Chaetomium globosum* (DSM 1962) was obtained from the German Collection of Microorganisms and Cell Cultures GmbH (Braunschweig, Germany).

The cultures were incubated for two weeks on potato dextrose agar to obtain spores from the mold. They were washed from the culture plates with peptone water from Lactan (Graz, Austria) and spore density was determined with a Neubauer cell counting chamber from Poly-Optik GmbH (Bad Blankenburg, Germany).

Wheat/rye/oat sourdough toast bread (9 × 9 cm squares) and white wheat toast bread were purchased at a local supermarket in Wels (Austria). They contained ascorbic acid and sodium acetate as preservatives and were precut. Sliced wheat/rye bread, which was purchased at a supermarket (Wels, Austria), and the whole loaf wheat/rye bread, were plain without seasonings and contained yeast and sourdough. The gluten-free corn bread was a sourdough bread. All whole loaf breads were purchased at local bakeries in Wels (Austria) and transported to the laboratory in a clean paper bag. The sliced samples were all packed in a sealed plastic bag.

Pictures were taken before analyses and after mold growth with a Samsung galaxy A33 5G smartphone camera (Version 13.1.00.77; 16:9 full HD 1920 × 1080 (72 dpi)).

### 2.2 Water content and pH of bread

The water activity (aw-value) was determined using a Lab Master – aw device from Novasina (Lachen, Switzerland), pH was determined by using a pH 7110 (Xylem Weilheim, Germany) and dry matter was measured using a Sartorius MA160 infrared moisture analyser (Göttingen, Germany).

### 2.3 Inoculation of bread with fungi and aliquoting

For all sliced bread samples, three slices were selected and whole loaves were cut into 4 cm pieces. The samples were stored in sterile plastic containers. On each sample, the inoculation site was marked with a blue, water insoluble dot on the top of the bread. Subsequently, 10,000 spores were inoculated onto the markings. The bread was incubated in the dark at 23 °C for 7 or 14 days in a sterile plastic container to prevent contamination from the environment. After *Aspergillus niger* reached a diameter of 6–7 cm, which was dependent on the bread type, the samples were collected. As the toast bread samples contained preservatives, the toast samples were incubated for 2 weeks, and all other bread types were stored for 1 week. For all bread types and all bread slices, a 28 mm^2^ aliquot was taken from the inoculation point and from an unaffected area of the same slice located 3 cm from the outer edge of the mold contamination. For the whole loaf bread types, 0.5 cm of the top and bottom of the slice were aliquoted at and below the inoculation spot and at 3 cm from the outer edge of the mold spot. The aliquots were weighted before storing at −20 °C until mycotoxin determination was performed.

### 2.4 Multispectral observation of mold growth on bread

At the end of each incubation period, selected bread samples were imaged using the VideometerLab (Herlev, Denmark), to observe mold growth not visible to the naked eye. The VideometerLab is a multispectral imaging tool that combines 19 different wavelengths between 365 nm and 970 nm. The white sphere contains numerous light-emitting diodes (LEDs) for each wavelength inside and a monochrome camera on top to record the data.

The bread samples were placed in a plate under the sphere, which was lowered onto the bread. Multispectral images were taken with a resolution of approximately 30 μm per pixel. Each pixel captured by the camera represents a surface reflection, enabling the determination of the texture, colour, and differences in chemical composition and therefore the fungal growth.

The process of transforming of multispectral images (MSI) involved performing normalized canonical discriminant analysis (nCDA) to minimise chromatic aberrations on the mold and determine the mold area for each image. First, each moldy spot was identified and marked as a region of interest (ROI), while the remaining areas were identified as bread background. A simple threshold was used to discriminate mold and background. Finally, the mold areas were displayed for specific bread samples.

### 2.5 Extraction of undesired growing mold samples

Additional mold spots were observed on both whole loaf bread samples after cutting and inoculation with the respective test species after one week. For DNA barcoding, spores were transferred from the moldy spot to a potato dextrose agar plate and incubated at 25 °C for one week. A 0.5 g bread aliquot was taken with a sterile plunger, transferred to a 1.5 ml reaction vessel, and stored at −20 °C until mycotoxin analysis.

### 2.6 DNA barcoding of undesired mold

Sequencing of the internal transcribed spacer region (ITS), the beta-tubulin sequence (Btub), and the sequences for calmodulin (CMD) and translation elongation factor 1 alpha (EF1) was used to identify unknown mold species.

The Phusion High Fidelity kit from New England Biolabs (Ipswich, Massachusetts, United States) was used for PCR analyses. The amount of 0.14 ng DNA per 20 μl reaction was used. All other concentrations were taken as recommended by the manufacturer.

A touchdown PCR was performed in a Bio-Rad CFX96 gradient cycler (Feldkirchen, Germany) under the following conditions: initial denaturation at 95 °C for 3 min, followed by 5 cycles with 95 °C denaturation for 30 s, annealing at 72 °C for 30 s and extension at 72 °C for 60 s. A second section with 5 cycles enriched the regions of interest with 95 °C denaturation for 30 s, annealing at 69 °C for 30 s and extension at 72 °C for 60 s followed by 30 cycles with 95 °C denaturation for 30 s, annealing at 50 °C for 30 s and extension at 72 °C for 60 s. The final extension step was performed for 7 min at 72 °C.

Subsequent agarose gel electrophoresis was performed by mixing 4 μl of sample and 2 μl of Midori Green® from Biozym Scientific GmbH (Hessisch Oldendorf, Germany) and by loading it on a 1.5% gel. Thermo Scientific Fast Ruler low range (Waltham, Massachusetts, Unites States) served as reference. Remaining PCR products were purified using the HiYield Gel/ PCR DNA Fragment Extraction Kit (Gauting, Germany) and sent for Sanger sequencing to Microsynth (Balgach, Switzerland). Sequence alignment was performed by the aim of the Basic Local Alignment Search Tool from the National Center for Biotechnology Information (Bethesda, Maryland, United States). Results were listed according to e-values. When e-values were equal, the percent identity was used as a second parameter. For all percentage identities below 100%, the sequences were checked individually for uncertain nucleotide identification.

### 2.7 Mycotoxin quantitation

The samples were prepared according to the protocol previously published. As several hundreds of compounds are included in the method, this results in a very large table. The related information is given open access in the supplementary information (Sulyok et al., 2020). Approximately, 1 g of sample was extracted with the extraction solvent (acetonitrile/water/acetic acid 79:20:1, *v*/v/v) for 90 min using a GFL 3017 rotary shaker (Burgwedel, Germany) and subsequently centrifuged for 2 min at 3000 rpm (radius 15 cm) on a Beckman Coulter Inc. GS-6 centrifuge (Fullerton, CA). The amount of the extraction solvent was used in the ratio of 1:4 of the weighted sample (i.e. 1 g of sample was extracted with 4 ml of extraction solvent). A 0.5 ml of extract was transferred into a glass vial and diluted with the same volume of dilution solvent (acetonitrile/water/acetic acid 20:79:1, v/v/v).

After appropriate mixing, 5 μL of the diluted extract was injected into a QTrap 5500+ MS/MS system (Sciex, Foster City, CA, USA) equipped with a TurboV electrospray ionization (ESI) source was coupled to an Agilent 1290 series UHPLC system (Waldbronn, Germany). Chromatographic separation was performed at 25 °C on a Gemini C18-column, 150 × 4.6 mm i.d., 5 μm particle size, equipped with a C18 security guard cartridge, 4 × 3 mm i.d. (both Phenomenex, Torrance, CA, USA).

Elution was carried out in binary gradient mode with a flow rate of 1000 μl/min. Both mobile phases contained 5 mM ammonium acetate and were composed of methanol/water/ acetic acid 10:89:1 (*v*/v/v) and 97:2:1 (v/v/v), respectively. Compound dependent LC-MS/MS parameters were published previously (Sulyok et al., 2020).

For confirmation of a positive identification, the ion ratio must agree with the related values of the standards within 30% as stated in EC guidance document on mycotoxin identification in food and feed (EU COMMISSION, 2017), whereas for the retention time, a stricter in-house criterion of ±0.03 min is applied.

Data were evaluated using the software Analyst 1.7.1 and Multiquant 3.0.3 The current LC-MS/MS method has been successfully validated for many complex food and feed matrices (Malachová et al., 2014; Steiner et al., 2020; Sulyok et al., 2020). The accuracy of the method is verified on a continuous base by participation in a proficiency testing scheme organized be BPEA (Genneviliers, France) with a current rate of satisfactory results of −2 < z < 2 of >96%.

The repeatability of the method determined on technical replicates is usually in the range of a few percent and the expanded measurement uncertainty of the method as determined by long-term performance on proficiency testing has been determined to be 50% with a coverage factor of 2 (Stadler et al., 2018).

Bread may contain traces of mycotoxins that originate from the flour used for bread production. Depending on weather and humidity, grain can become moldy on the field and the produced mycotoxins are then detectable in the final product, such as bread. To know the background contamination of the bread before the artificial inoculation, all bread samples were therefore sampled on three independent spots and the presence of mycotoxin was measured in triplicates.

### 2.8 Statistics

Graphs were generated using GraphPad Prism (GraphPad Software, San Diego, CA, USA; version 8.0.2) and Corel Draw X8 SE (Corel Corporation, Ottawa, Canada). Sequences were aligned in the Basic Local Alignment Search Tool for Nucleotides provided by the NIH (BLASTN; BLAST: Basic Local Alignment Search Tool (nih.gov)). Short sequence alignments were performed with GENtle (Version 1.2, November 2002, Free Software Foundation, Inc., Boston, United States).

Multispectral images were interpreted with VideometerLab Software version 3.22 (Herlev, Denmark). The obtained multispectral images were transformed using normalized canonical discriminant analysis (nCDA).

## 3 Results and discussion

### 3.1 Mycotoxin content of bread before inoculation

The wheat/rye/oat toast and the wheat/rye whole loaf bread were free of metabolites. Traces of citrinin were found in the white toast (8 ng/kg) and in the sliced wheat/rye bread (6 ng/kg). The gluten free corn bread contained viriditoxin (260 ± 50 μg/kg) and deoxynivalenol (47 ± 5 μg/kg). As they can withstand heat treatment such as baking, significant amounts of the substances are not destroyed (Dropa et al., 2014; Giménez et al., 2013; Heidari et al., 2014; Milani & Heidari, 2017).

There is no tolerable daily intake level for citrinin, because no authority can guarantee the safety of this substance below a certain limit, but there is a level of no concern for nephrotoxicity of 0.2 μg/kg body weight for citrinin per day suggested by EFSA (EFSA, 2012). Therefore, a 70 kg person could eat 1700 kg of the white toast bread and still be below the exposure limit for nephrotoxicity. For viriditoxin, a lethal dose 50% value for mice of 2.8 mg/kg intraperitoneal is published (Wong & Hamill, 1976), which would correspond to several hundred corn breads for the 70 kg person. The tolerable daily intake for deoxynivalenol is 1 μg/kg body weight per day (Knutsen et al., 2017), which is equivalent to an acceptable consumption of slightly >1 kg of corn bread. The guideline values only refer to single contaminations and there are no regulations on the effect of a combination of mycotoxins (Alassane-Kpembi et al., 2017; Smith et al., 2016).

### 3.2 Water content has impact on mold growth

Toast bread samples had the lowest water activity (0.925 and 0.877) and the highest dry matter (62% and 63%) during inoculation. Growth ability of mold was reduced on toast containing preservatives, and therefore the toast samples were incubated for two weeks to achieve the same size of mold as on the other bread types, which were incubated for one week. The pH values varied between 4.41 (wheat/rye whole loaf) and 5.25 (wheat/rye sliced with yeast). The gluten free corn bread, which had the highest aw value and lowest dry matter, developed fungal growth even on non-inoculated spots. Measurements of pH, aw and dry matter were performed only on the day of inoculation (Table 1).

**Table 1:** Physical parameters of the bread types used in this study.

	Wheat/rye/oat sourdough toast slices		white wheat toast with yeast sliced		sliced wheat/rye bread with yeast		wheat/rye sourdough whole loaf		Gluten-free whole corn bread loaf free of yeast	
	mean	STD Dev	mean	STD Dev	mean	STD Dev	mean	STD Dev	mean	STD Dev
pH	5.06	0.05	5.03	0.02	5.25	0.02	4.41	0.02	4.84	0.01
aw	0.93	0.02	0.878	0.01	0.931	0.01	0.94	0.00	0.95	0.00
(%) dry matter	62.49	1.84	63.24	1.63	53.06	1.15	51.79	0.68	48.60	1.60

The physical parameters were very similar and did not result in any difference in the mold's ability to grow.

### 3.3 Mold growth and mycotoxin distribution

**Fig. 1:**
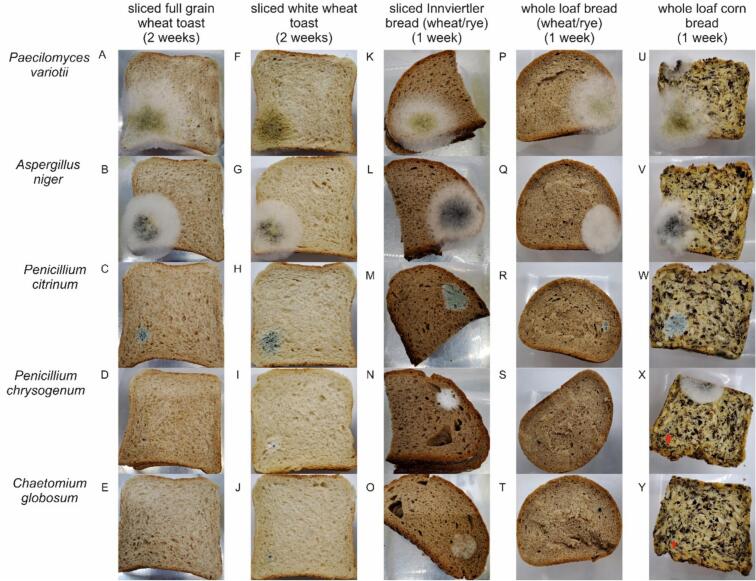
Five types of bread were inoculated with five strains of mold. The inoculation spot was labeled with a blue dot and was highlighted in two pictures (X,Y) with red arrow, as the colour pattern of the corn bread makes it difficult to see. The bread types used in this study were wheat/rye/oat sourdough toast bread (A - E), white wheat toast (F - J), sliced wheat/rye bread (K - O), whole loaf wheat/rye bread (P - T), and gluten-free corn bread (U - Y). The used mold strains were *Paecilomyces variotii* (A, F, P, K, U), *Aspergillus niger* (B, G, L, Q, V), *Penicillium citrinum* (C, H, M, R, W), *Penicillium chrysogenum* (D, I, N, S, X), and *Chaetomium globosum* (E, J. O, T, Y). (For interpretation of the references to colour in this figure legend, the reader is referred to the web version of this article.)

Mold spots of *Paecilomyces variotii*, *Aspergillus niger*, *Penicillium citrinum* and *Penicillium chrysogenum* appeared on every inoculation spot (blue dot) on the first layer except for *Penicillium chrysogenum* on wheat/rye/oat toast and whole loaf bread (Fig. 1: D, S). *Paecilomyces variotii* showed slightly stronger growth ability than the others on the two toast samples (Fig. 1: A, F). *Chaetomium globosum* showed no growth within the incubation time except for sliced wheat/rye bread (Fig. 1:N). VideometerLab analyses confirmed that mold grew weakly on the respective *Chaetomium globosum* inoculation spots (Supplementary Fig. 1: C, D) and *Penicillium chrysogenum* on white toast and wheat/rye/oat toast (Supplementary Figure1: A, B). To ensure that sufficient viable spores were used for inoculation, the same amount of spores was also applied to agar plates, which confirmed significant growth within a few days at 30 °C.

The mold on the three bread types grew much faster than the mold spots on the toast types, which can be explained by the presence of the preservatives ascorbic acid and sodium acetate in both toast types.

### 3.4 Metabolites determined in the inoculation spots and in respective distances

**Fig. 2:**
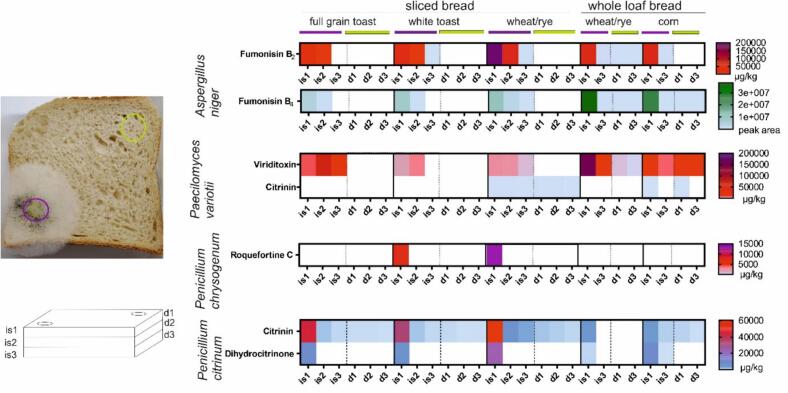
Heat map of mycotoxin distribution throughout the bread samples. The inoculation spots (purple) are labeled with ‘is’ and the spots in 3 cm distance (green) are labeled with ‘d’. The numbers indicate the layer of the bread. Results are subdivided into quantified mycotoxins (red and blue) and those with peak area data (green and blue). Each box represents one bread inoculated with one mold strain. Whole wheat/rye and corn bread have two sampling spots, as they consist of just one 4 cm large slice of the whole bread. (For interpretation of the references to colour in this figure legend, the reader is referred to the web version of this article.)

The metabolites quantified in our intentionally mold spots were fumonisin B_2_, citrinin, dihydrocitrinone, roquefortine C, and viriditoxin. Fumonisin B_4_ was only identified but not quantified due to the lack of the analytical standard. Therefore, only relative amounts can be reported for fumonisin B_4_. Metabolites indicating the presence of *Chaetomium* were expected but not detected in this study. This may be due to weak growth of the fungus on the five bread types used in this study. In a previous paper, the ability of *Chaetomium globosum* to produce mycotoxins on bread was proven (Ollinger et al., 2022). Only the mycotoxins produced by the inoculum species are presented in Fig. 2. The substances that were already present in the bread before treatment are not shown in this Figure for clarity.

The inoculation spots showed the highest concentration of mycotoxins in the upper layer (is1) which decreased with each layer (is2 – is3). For example, fumonisin B_2_ in the full grain toast with *Aspergillus niger*, the concentration in the inoculation spot was 23,050 μg/kg and was reduced in the second slice to a value of 7640 μg/kg. In the third slice under the inoculation spot and in all samples measured at 3 cm distance, no fumonisin B_2_ was detectable. *Penicillium citrinum* produced 45,100 μg/kg citrinin in the first slice of the inoculation spot. In the second slice, the concentration dropped to 840 μg/kg and in the third slice, the concentration was 270 μg/kg. In the three reference spots at a distance of 3 cm, 100–130 μg/kg were determined for citrinin. In the corn bread, for example, the amount of citrinin was 1990 μg/kg in the inoculation spot and beneath the inoculation spot on the other side of the 4 cm thick slice, the concentration was 500 μg/kg. On the upper side at 3 cm distance, 410 μg/kg citrinin were determined and in the same distance but on the other side of the bread slice, 100 μg/kg. An exception was viriditoxin, because in all toast samples inoculated with *Paecilomyces variotii*, the concentration was higher in the second slice. The full grain toast had 6620 μg/kg viriditoxin in the upper slice, which was the inoculation spot. The concentration then increased in the second slice to 62,190 μg/kg and to 13,420 μg/kg in the third slice. The white toast contained 2900 μg/kg viriditoxin in the inoculation spot and 4800 μg/kg in the second slice.

In most samples, at 3 cm distance (d1-d3), the bread was less affected by toxins than at the inoculation spot. *Aspergillus niger* produced the typical metabolites such as fumonisins in varying concentrations on all types of bread. The lowest concentrations were detected in toast breads. *Paecilomyces variotii* produced viriditoxin and citrinin. The toast samples were less affected of mycotoxins and not detectable at 3 cm distance from the mold spot, although the growth behavior was particularly strong in these samples. The *Penicillium chrysogenum* mycotoxin roquefortine C was only detected in the inoculation spots of white toast and sliced wheat/rye bread. *Penicillium citrinum* produced detectable amounts of citrinin in all regions of all bread samples with the highest concentrations in the inoculation spots except for the whole loaf wheat/rye bread. Dihydrocitrinone was present at each inoculation spot. In corn bread, dihydrocitrinone had a concentration of 9260 mg/kg at the inoculation spot and was detectable also 4 cm beneath the inoculation spot with 30 μg/kg. The overall mycotoxin load was highest in sliced wheat/rye bread and in corn bread.

Unfortunately, with the current state of knowledge and the respective legislation, it is not possible to draw any valid conclusions about the safety of moldy breads, because there are no clear guidelines like tolerable daily intake values and there are still uncertainties due to limited amount of information. For example, the CONTAM Panel concluded that there is no concern for nephrotoxicity in humans at an exposure level of 0.2 μg/kg body weight for citrinin per day (EFSA, 2012), but still, they see too many uncertainties to derive a mandatory tolerable daily intake value. Checking the measured values for citrinin in the inoculation spots of *Penicillium citrinum* on the bread type wheat/rye sliced bread being 60.700 μg/kg, it can be calculated that a 70 kg person would exceed the value >40 times if the person would eat just 1 g of the inoculation spot of moldy bread. On the very same slice in three centimeters distance from the moldy spot, 790 μg/kg were measured, which corresponds to 17 g of contaminated bread that can be eaten to reach a level of no concern for nephrotoxicity. On slice three, a value of 300 μg/kg was determined, which means that 50 g of this contaminated region can be eaten to reach the mentioned exposure level. Therefore, it is not advisable to eat partially moldy bread, as a normal portion of bread contains >50 g.

Official maximum levels in bread are only available for zearalenone (50 μg/kg) with a TDI of 0.25 μg/kg body weight per day and deoxynivalenol (500 μg/kg) with a TDI of 1 μg/kg body weight per day, while zearalenone was not found in any of the bread samples and deoxynivalenol was present in all corn bread samples, but well below the regulatory limit (EFSA, 2016; EU COMISSION, 2023; Knutsen et al., 2017). Providing a well-founded statement about bread safety is not possible as the lethal dose 50% (LD50) or tolerable daily intake (TDI) reference values are not available for most of the fungal compounds. Official maximum levels in bread are available only for zearalenone (50 μg/kg) with a TDI of 0.25 μg/kg body weight per day and deoxynivalenol (500 μg/kg) with a TDI of 1 μg/kg body weight per day, while zearalenone was not found in any of the bread samples and deoxynivalenol was present in all corn bread samples, but well below the regulatory limit (EFSA, 2016; EU COMISSION, 2023; Knutsen et al., 2017). Due to the high number of varying metabolites, it is not possible to clearly determine the safety of contaminated bread, also because bread in many cases contains mycotoxins derived from affected grains (Saladino et al., 2017; Vaclavikova et al., 2013).

### 3.5 Inadvertent contamination spots on bread

**Fig. 3:**
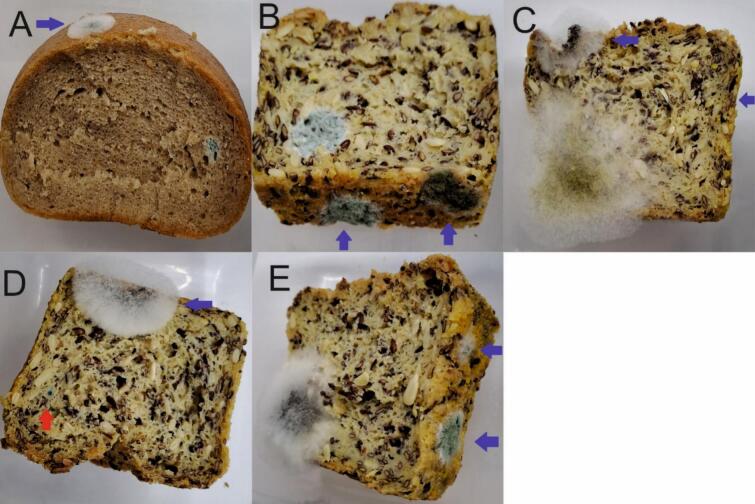
Inadvertent contaminations of cut whole loaves indicated with blue arrows. (A) *Aspergillus pseudoglaucus* on whole loaf wheat/rye bread inoculated with *Penicillium citrinum* in the right lower corner, (B) *Penicillium crustosum* (left) and *Cladosporium ramotenellum* on whole loaf corn bread inoculated with *Penicillium citrinum* (inner side), (C) *Aspergillus tubingensis* (left upper corner) and *Penicillium bialowiezense* (side) on whole loaf corn bread inoculated with *Paecilomyces variotii* (left lower corner), (D) *Aspergillus tubingensis* on whole loaf corn bread inoculated with *Penicillium chrysogenum* (red arrow) and (E) *Penicillium paxilli* (upper arrow) and *Penicillium crustosum* (lower arrow) on whole loaf corn bread inoculated with *Aspergillus niger* (left lower corner). (For interpretation of the references to colour in this figure legend, the reader is referred to the web version of this article.)

Six undesired mold spots, that are indicated by blue arrows in Fig. 3, were observed on two types of bread and a total of five samples. An aliquot was taken for metabolite profiling and a smear test was taken for identification by DNA barcoding (Table 2). On the wheat/rye whole loaf bread, *Aspergillus pseudoglaucus* could be identified and on the whole loaf corn bread, *Penicillium crustosum*, *Cladosporium ramotenellum*, *Aspergillus tubingensis*, *Penicillium bialowiezense*, and *Penicillium paxillin* were growing.

**Table 2:** DNA barcoding for undesired mold species identification.

bread	initial inoculation strain	identified species	Btub	CMD	ITS1F/4	ITS34	EF1	Btub	CMD	ITS1F/4	ITS34	EF1
whole loaf wheat/rye	*Penicillium citrinum*	***Aspergillus pseudoglaucus***	472/472	939/939				100	99.8	100	100	
corn bread	*Penicillium citrinum*	***Penicillium crustosum***	442/442	457/457				96.86*	98.43*			
corn bread	*Penicillium citrinum*	***Cladosporium ramotenellum***			942/942	472/472	1413/1413				100	100
corn bread	*Paecilomyces variotii*	***Penicillium bialowiezense***	466/466	472/472	942/942	466/466		100	100	100	99.61	
corn bread	*Paecilomyces variotii*	***Aspergillus tubingensis***	942/942	937/937		268/268		100	99.8		84.29	
corn bread	*Penicillium chrysogenum*	***Aspergillus tubingensis***	942/942	935/935		472/472		100	100			
corn bread	*Aspergillus niger*	***Penicillium paxilli***	900/900	472/472				100	100			
corn bread	*Aspergillus niger*	***Penicillium crustosum***	442/442	472/472				96.86*	96.86*			
								* uncertain nucleotide				

The query cover in the database alignments was always 100% with except for one *Aspergillus tubingensis* sequence being 99%. The percentage of sequence identity was almost 100%, except for a few sequences with uncertain nucleotides in the sequenced strain (Table 2) indicated with an asterisk. Without uncertain nucleotides, all sequences would be 100% identical. The aligned sequences ranged in length from 268 bp to 1413 bp.

The lowest percent identity of 96.86% was obtained for the *Penicillium crustosum* beta-tubulin sequence containing eight mismatches due to inaccuracies at these positions in a 442 bp long sequence. For all sequences without inaccurate identifications, the percent identity was 100%.

The shortest sequence length was 261 bp for *Aspergillus tubingensis* EF1 and the longest sequence was 1413 bp for *Cladosporium ramotenellum* in the ITS region.

It is most likely that the species originated from the producing bakery or the packaging, as the bread was immediately transferred to the laminar hood in the laboratory in the packaging after purchase. The loaves were handled using sterile equipment and stored in autoclaved plastic containers. Further, the identified mold types are not available in the laboratories mold library, except for *Cladosporium ramotenellum*, which, however, was not cultivated in this lab within the last year.

### 3.6 Mycotoxins on the inadvertent contaminated bread samples

**Fig. 4:**
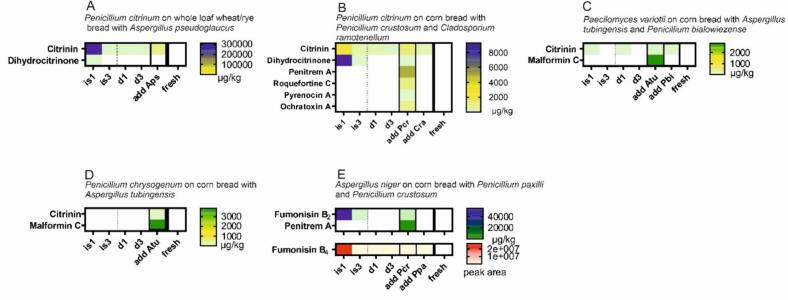
Mycotoxin load of inadvertently contaminated bread samples in comparison with the intentionally mold metabolite profile at the inoculation spot (is1,3) and at distance (d1,3). (A) Wheat/rye whole loaf sample inoculated with *Penicillium citrinum* and an additional spot of *Aspergillus pseudoglaucus* (add Aps), (B) Corn bread with inoculated *Penicillium citrinum* and additional spots of *Penicillium crustosum* (add Pcr) and *Cladosporium ramotenellum* (add Cra), (C) corn bread with intentionally contaminated *Paecilomyces variotii* and additional *Aspergillus tubingensis* (add Atu) and *Penicillium bialowiezense* (add Pbi), and (D) corn bread with *Aspergillus niger* and inadvertently grown *Penicillium paxilli* (add Ppa) and *Penicillium crustosum* (add Pcr). The metabolite profile of the untreated sample (fresh) is shown in the last column for both bread types, which is empty in all cases.

The undesired growing mold spots showed specific metabolite patterns, typical of the respective strains (Fig. 4). Roquefortine C was found in *Penicillium* spots. *Aspergillus* species produced malformin C. Although the content of the intentionally induced mycotoxins decreased with distance, higher concentrations of the same were detected in the undesired contamination spots compared to the unaffected regions (d1–3). Citrinin concentrations were higher in samples of bread with at least one second strain present, and especially in the spot of the undesired mold, the citrinin concentration was increased. This could be the result of some kind of competition between the two or more species. Due to limited data, this cannot be clearly proven and may be an interesting topic for further research.

The presence of citrinin in foodstuff like wheat, barley, rye, flour, bread, pasta, beans, and juices has already been demonstrated (EFSA, 2012; López Sáncheza et al., 2017), but there is still a lack of knowledge on the causes of its production and why it is sometimes produced in such large quantities.

The high number of inadvertently occurring mold spots in the corn bread and the initial high mycotoxin load most likely, indicate the use of contaminated ingredients for bread production. The bread was wrapped in clean and protective materials and immediately placed in the laminar hood in the laboratory, so it can be concluded that spores from the bakery plant, the distributor or from the indoor air in the bakery were deposited on the surface of the bread and led to mold growth.

## 4 Conclusion

The hypothesis in this paper was that the mycotoxin release would be limited to the immediate area of the visible mold growth. This was true for most of the metabolites, except for citrinin, which was detectable in almost all measured areas of the inoculated samples. The initial mycotoxin background in the loaves contained viriditoxin, deoxynivalenol and traces of citrinin. These metabolites originate from Penicillia and Fusaria, which may have already been present in the field, due to unfavorable conditions. Avoiding mold on bread in common households could be achieved by optimized storage conditions, and especially by an increased awareness of consumers regarding the amount of purchased goods that are really required.

The disposal of large quantities of bakery products is already a problem in the food industry and strategies are required to reduce the food waste. One attempt would be to use aged but uncontaminated bread as feed for farm animals, where mycotoxins must be avoided too, due to safety and health issues for the animals. Another recommendation is to conduct more research on the production of mycotoxins and fungi and especially on the metabolism of mycotoxins in insects, which could be utilized as feed or potentially even as food. Moreover, the results on mycotoxin production and diffusion of these secondary metabolites in food products should be communicated beyond the scientific community due. The fact that mycotoxins, such as citrinin, tend to spread throughout the entire food product, whereas others like roquefortine C remain near in close vicinity of inoculation site is of potential interest for the food industry and bakeries.

## CRediT authorship contribution statement

**Nicole Ollinger:** Writing – original draft, Methodology, Investigation, Data curation, Conceptualization. **Alexandra Malachova:** Writing – review & editing, Methodology, Investigation, Data curation. **Michael Sulyok:** Methodology, Investigation. **Rudolf Krska:** Funding acquisition, Conceptualization. **Julian Weghuber:** Writing – review & editing, Funding acquisition, Conceptualization.

## Declaration of competing interest

The authors declare that they have no known competing financial interests or personal relationships that could have appeared to influence the work reported in this paper.

## Data Availability

Data will be made available on request.

## Appendix A Supplementary data

Figure 1A: Examples of inoculated bread where no fungal growth was visible to the naked eyes. Normalized canonical discriminant analysis proved minimal growth of *Penicillium chrysogenum* on white toast (A) and wheat/rye/oat toast (B), and *Chaetomium globosum* on corn bread (C) and whole loaf wheat/rye bread (D). Image 1
