# Supplementary material for: Mycotoxin contamination in moldy slices of bread is mostly limited to the immediate vicinity of the visible infestation
Source: Food Chem X. 2024 Jun 18;23:101563. doi: 10.1016/j.fochx.2024.101563 (PMC11231700; doi:10.1016/j.fochx.2024.101563)
Supplement: Supplementary file 1 — Figure 1A: Examples of inoculated bread where no fungal growth was visible to the naked eyes. Normalized canonical discriminant analysis proved minimal growth of Penicillium chrysogenum on white toast (A) and wheat/rye/oat toast (B), and Chaetomium globosum on corn bread (C) and whole loaf wheat/rye bread (D). [file mmc1.docx]

Supplementary information

**Mycotoxin contamination in moldy slices of bread is mostly limited to the immediate vicinity of the visible infestation**

Nicole Ollinger^a*^, Alexandra Malachova^b^, Michael Sulyok^c^, Rudolf Krska^b,c,d^, Julian Weghuber^a,e*^

^a^ FFoQSI – Austrian Competence Centre for Feed and Food Quality, Safety & Innovation, Stelzhamerstr. 23, 4600 Wels, Austria

^b^ FFoQSI – Austrian Competence Centre for Feed and Food Quality, Safety & Innovation, Konrad Lorenz Str. 20, 3430 Tulln, Austria

^c^ Department for Agrobiotechnology (IFA-Tulln), University of Natural Resources and Life Sciences, Vienna (BOKU), Konrad Lorenz Str. 20, 3430 Tulln, Austria

^d^ Institute for Global Food Security, School of Biological Sciences, Queen’s University Belfast, University Road, Belfast, BT7 1NN, Northern Ireland, United Kingdom

^e^ University of Applied Sciences Upper Austria, Stelzhamerstrasse 23, 4600 Wels, Austria.


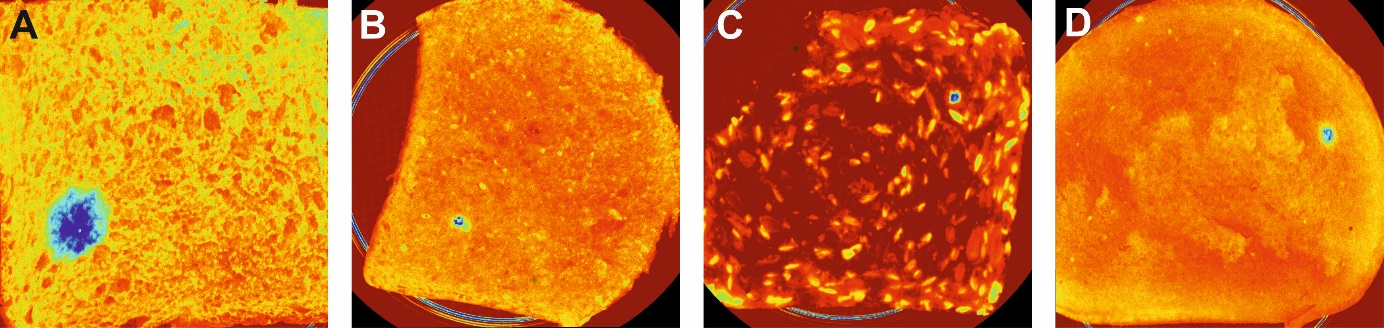


Supplementary Figure 1: Examples of inoculated bread where no fungal growth was visible to the naked eyes. Normalized canonical discriminant analysis proved minimal growth of *Penicillium chrysogenum* on white toast (A) and wheat/rye/oat toast (B), and *Chaetomium globosum* on corn bread (C) and whole loaf wheat/rye bread (D).
